# Vaccinomics and Adversomics in the Era of Precision Medicine: A Review Based on HBV, MMR, HPV, and COVID-19 Vaccines

**DOI:** 10.3390/jcm9113561

**Published:** 2020-11-05

**Authors:** Jasna Omersel, Nataša Karas Kuželički

**Affiliations:** Faculty of Pharmacy, University of Ljubljana, Aškerčeva cesta 7, 1000 Ljubljana, Slovenia; jasna.omersel@ffa.uni-lj.si

**Keywords:** adversomics, COVID-19, genetics, hepatitis B, human Papilloma virus, MMR vaccine, precision medicine, vaccinomics

## Abstract

Precision medicine approaches based on pharmacogenomics are now being successfully implemented to enable physicians to predict more efficient treatments and prevention strategies for a given disease based on the genetic background of the patient. This approach has already been proposed for vaccines, but research is lagging behind the needs of society, and precision medicine is far from being implemented here. While vaccinomics concerns the effectiveness of vaccines, adversomics concerns their side effects. This area has great potential to address public concerns about vaccine safety and to promote increased public confidence, higher vaccination rates, and fewer serious adverse events in genetically predisposed individuals. The aim here is to explore the contemporary scientific literature related to the vaccinomic and adversomic aspects of the three most-controversial vaccines: those against hepatitis B, against measles, mumps, and rubella, and against human Papilloma virus. We provide detailed information on the genes that encode human leukocyte antigen, cytokines and their receptors, and transcription factors and regulators associated with the efficacy and safety of the Hepatitis B and Measles, Mumps and Rubella virus vaccines. We also investigate the future prospects of vaccinomics and adversomics of a COVID-19 vaccine, which might represent the fastest development of a vaccine ever.

## 1. Introduction

Despite some initial skepticism, it is undeniable that the era of precision (or personalized) medicine is here, as formalized by the launch of the Precision Medicine Initiative in 2015 [1]. The paradigm that one size does not fit all is slowly taking hold in the minds of clinicians, and also of patients, and by society as a whole. What is more, we are starting to realize that personalized approaches might be a solution to the public distrust in medicines, pharmaceutical companies, and science in general that has emerged over the last few decades, and mainly in developed countries.

One of the first fields of precision medicine that developed was pharmacogenomics, which provided both the scientific basis and clinical outcomes that have together promoted the practice of personalized drug therapies; i.e., prescribing each patient with the right dose of the right drug, thus maximizing the efficiency and safety of the therapy [2]. The application of this same principle to vaccines has been named vaccinogenomics, or vaccinomics. The term vaccinomics was first used by Hoffman et al. in 1998 [3] and extensively studied by Gregory A. Poland and the Mayo Clinic Vaccine Research Group [4]. By definition, vaccinomics explores the influence of genetic (and non-genetic) factors on the heterogeneity of vaccine-induced immune responses between individuals and populations [5]. While vaccinomics has been more focused on vaccine effectiveness, the field of adversomics was again first introduced by Poland, this time in 2009, and this is more concerned about the side effects of vaccines [6].

As both of these fields are at most a decade old, relatively little research has been carried out here compared to pharmacogenomics. Interestingly, most of the studies in the field of personalized vaccinology have investigated vaccine effectiveness, with only a few relating to vaccine-induced side effects. This is demonstrated by the number of PubMed papers on vaccinomics and adversomics over the last 10 years to date, at 334 and 65, respectively. While vaccine effectiveness has been studied for most of the existing vaccines [2], adversomic studies have mainly been carried out for the vaccines against smallpox (Variola virus) and measles, mumps, and rubella (MMR) viruses [7]. Although vaccine effectiveness is indeed a very important issue, especially from the financial point of view, the adversomics of vaccines also need to be developed further as they hold great potential for the resolving of public fears and concerns about vaccine safety, which is the cause of the decreased vaccination rates in developed countries.

Many false side effects have been attributed to vaccines by pseudoscience and fraudulent scientists (such as autism and the MMR vaccine, by Andrew Wakefield). This has caused tremendous harm to vaccination programs throughout the world. However, while vaccines are not without risk, sadly, the public hysteria about false vaccine risks often overshadows the challenges of detecting the real risks [8]. Vaccines face more strict safety standards than other pharmaceutical products as they are given to healthy populations of mostly children to prevent diseases. However, at the same time, due to the high effectiveness of vaccination programs, the target diseases and the consequences that the vaccines protect from are not generally apparent to the general public any more [8]. This can thus give the impression that vaccines are no longer necessary, while they can potentially cause harm. What the public does not realize is that actual serious side effects, which are extremely rare, are unlikely to be detected in the pre-licensing phases of vaccine testing, as the number of test subjects is simply too small; i.e., the power of the study is too low. In other words, pre-licensing studies can provide excellent estimates of efficiency, but they cannot rule out any very rare side effects [8,9]. This is demonstrated by three notable cases of vaccine retraction due to side effects that were revealed in the post-licensing phase, which have received surprisingly little public attention [8]: (i) a rotavirus vaccine from Wyeth Lederle (RotaShield) was retracted in 1999 for causing intussusception (a very rare but serious side effect) in infants; (ii) in 2007, it was found out that infants immunized with the combined MMR plus Varicella virus vaccine resulted in a greater risk of febrile seizures compared to those who received the conventional MMR vaccine; and (iii) after extensive vaccination of almost the entire populations of Finland and Sweden with the GlaxoSmithKline H1N1 vaccine Pandemrix, a steep increase in the incidence of the otherwise very rare autoimmune disease of narcolepsy was seen. After extensive investigations, the European Medicines Agency (EMA) confirmed an association between the GlaxoSmithKline H1N1 vaccine and narcolepsy, which was later also detected in England, Ireland, France and Norway. However, the causal mechanisms remain unclear in this case [10].

The knowledge from the fields of vaccinomics and adversomics will be of the utmost importance in the process of the development and testing of the COVID-19 vaccines. A wide range of physiological and pathological responses to the infection with SARS-CoV-2, observed in different individuals, indicates that genetic factors play a key role in the modulation of the immune response to this virus. Thus, we might predict that the same genetic factors will be involved in the vaccine response. Consequently, the use of vaccinomics and adversomics in the vaccine design will facilitate the development of a safe and effective COVID-19 vaccine.

The aim of this report is to explore the contemporary scientific literature on the three most-controversial vaccines from the vaccinomic and adversomic aspects: those against Hepatitis B virus (HBV), MMR viruses, and human Papilloma virus (HPV).

## 2. Vaccinomics

The current vaccination schemes do not take into account interindividual differences in vaccine efficacy. Thus, all individuals are vaccinated with the same number of doses, even though some individuals might achieve seroconversion with a lower number of vaccine doses, while others might remain unresponsive even after administration of the standard number of doses. Therefore, prediction of vaccine efficiency before the administration of the vaccine to any particular individual might be useful for health and economic reasons. Individuals deemed to be unresponsive by vaccinomic tests could then be given a further vaccine dose while monitoring for seroconversion to assure that they are protected against the disease. On the other hand, hyper-responsive individuals might be vaccinated with a smaller number of doses, which would be beneficial from the economic point of view.

### 2.1. Vaccinomics of the Hepatitis B Virus Vaccine

Although seroconversion after three doses of the HBV vaccine is relatively high [11], inadequate production of protective antibodies has been observed for 4% to 10% of healthy individuals [12]. The number of non-responders is even higher in patients with autoimmune diseases [13,14]. Recently, the hypothesis has been proposed that HBV vaccine nonresponse might predict latent autoimmunity [15].

A relatively large number of vaccinomic studies have been conducted in connection with HBV, some of which were carried out even before the term vaccinomics was coined. However, the majority of these focused on HBV vaccine efficacy, i.e., predicting good versus poor responses in terms of seroconversion on the basis of genetic markers. The immune response to the HBV vaccine shows a high level of heritability (77%), as was determined in 207 twin pairs [16]. Early studies identified individuals as HBV vaccine poor responders who expressed high levels of the human leukocyte antigen HLA-DR3 and HLA-DR7 antigens (i.e., homozygous for haplotypes HLA-B8, *SC01*, *DR3*, *HLA*-*B44*, *FC31*, and *DR7*) [17,18]. In later studies, it was determined that the most important *HLA* locus for HBV vaccine response prediction was *HLA*-DRB1* [19] and that certain alleles at this locus are linked to nonresponse and others to good response to HBV vaccine. In several studies, alleles *HLA*-DRB1*0301, *HLA*-DRB1*0701, and *HLA*-DQB1*0201 were associated with no production of anti-HBV antibodies after vaccination (HBV vaccine nonresponse), while alleles *HLA*-DRB1*01, *HLA*-DRB1*11, and *HLA*-DRB1*15 were associated with rapid production of protective antibodies (HBV vaccine good response) [19,20]. Functional studies have revealed that in humans, the mechanism of nonresponse for the *HLA*-DRB1*0301 and *HLA*-DRB1*0701 alleles was due to the lack of specific T cells that recognized a particular major histocompatibility complex (MHC)/HBV surface antigen peptide complex, rather than due to the failure of the MHC molecule to bind the HBV surface antigen peptide, as shown in a mouse model [21].

As conventional HLA typing is complex and time-consuming, the use of single nucleotide polymorphisms (SNPs) as vaccinomic markers that can be defined using simple genotyping methods (e.g., TaqMan technology) would be more suitable for clinical implementation. Indeed, genome-wide association studies (GWAS) have detected SNPs in the HLA genes that are in line with the older studies mentioned above and have great potential to be used as vaccinomic markers. In GWAS on an Indonesian cohort, two SNPs in *HLA*-*DQB1* (*rs32734227*, *rs32734289*) were identified as markers of HBV vaccine nonresponse [22], while a genome wide association study (GWAS) on a Han Chinese population revealed *rs477515* at *HLA*-*DRB1* to be an independent marker of nonresponse to the HBV vaccine [23]. In a population of adult non-responders to infants given the HBV vaccine, a GWAS by Wu et al. detected a SNP at the *HLA*-*DPB1* locus (*rs7770370*) as a nonresponse marker to the HBV vaccine booster dose [24]. Recently, a GWAS on Japanese individuals identified *HLA* alleles of poor response to HBV vaccine as DRB1*0405, DQB1*0401, and DPB1*0501 [25]. As all of these GWASs were performed in Asian populations, the relevance of the genetic markers identified needs to be validated in Caucasians and Africans.

Apart from *HLA* loci, other genes have been shown to influence HBV vaccine responses. For example, null alleles of the complement component *C4A* gene (*C4AQ0*; due to deletion or non-expression) have been associated with poor response to the HBV vaccine [26]. Furthermore, Toll-like receptors (TLRs) and cytokines and their receptors have been identified as potential vaccinomics markers. The interleukin (*IL*)-*1β* haplotype *rs1143633* (A) + *rs1143627* (G) was associated with HBV vaccine non-response, as well as SNPs in genes encoding IL-13 (*rs1295686*) and IL-4 (*rs2243248*). Conversely, *rs1805015* for the IL-4 receptor (IL4RA) and *rs3804100* for TLR2 were associated with a good response to the HBV vaccine [27]. Several other loci have been connected to HBV vaccine nonresponse in different GWASs, although the studies will need replication before these can be conclusively considered as vaccinomic markers. Interestingly, a SNP in the butyrophilin like (*BTNL*)2 gene (*rs3763316*) was among the top hits in two independent GWASs [22,23], which indicates its potential as a useful vaccinomics marker. A recent GWAS on the Japanese population identified another SNP in the same gene (*rs4248266*) as a predictor of HBV vaccine response [25]. This gene encodes an MHC-II-associated, type I transmembrane protein. This is believed to be involved in immune surveillance, by serving as a negative T cell regulator through decreasing T cell proliferation and cytokine release. More details of the genes associated or believed to be associated with HBV vaccine responses are given in Table 1, Table 2, Table 3 and Table 4.

### 2.2. Vaccinomics of the MMR Vaccine

Despite the availability of a highly effective measles vaccine, a resurgence of measles has been seen across the developed world. Most of these cases can be attributed to non-vaccinated individuals (usually due to the fears spread by the antivaccine movements), although some of those infected had been fully vaccinated. Numerous studies have shown that host genetic factors result in inter-individual variations in measles-vaccine-induced antibodies, and hence can have a role in vaccine failure.

Heritability of an antibody response to measles vaccination was estimated to be 88.5% in a twin study [49] and 49% using the complete genetic information from a GWAS of 935 individuals [50]. This suggests that the inter-individual variations in antibody responses to measles vaccine arise through the combined small effects of many genes [50].

Early vaccinomic studies on the MMR vaccine mainly involved the genes that encode various cytokines and their receptors. As cytokines are important mediators of both adaptive and innate immune responses, it is not surprising that many SNPs in these genes were associated with responses to the MMR vaccine. Minor alleles of two polymorphisms in the tumor necrosis factor (*TNF*) (*rs1799964*) and *IL-6* (*rs2069849*) genes were associated with low rates of expression of measles-specific antibodies after the MMR vaccine in a Somali population [34], while polymorphisms in *rs2228149* in IL-2 receptor subunit α (*IL2RA*) and *rs1143634* in *IL-1B* have been associated with low rates of expression mumps-specific antibodies [34]. Even more polymorphisms have been identified as associated with the nonresponses to the rubella component of the MMR vaccine: *rs7873167*, *rs3885423*, *rs1364613* and *rs1364612* in interferon (*IFN*)*B1*; *rs12722713* and *rs12722698* in *IL2RA*; *rs228937* in *IL2RB*; *rs2069824* in *IL-6*; and *rs4149650* in ’TNF receptor superfamily member 1A’ (*TNFRSF1A*) [34]. At the same time, several polymorphisms of the *HLA* loci were addressed in association with the MMR vaccine response. Consistent associations with humoral responses to the measles component after two doses of MMR vaccine were shown in three independent cohorts for the following *HLA* alleles: B*3503 (good response), and DQA1*0201 and DRB1*0701 (poor responses) [29,30]. Distinct alleles of poor response were also detected for rubella and mumps components of the MMR vaccine: A*2705, A*5701, DPA1*0201, DPB1*0301, DPB1*1301 (rubella), and DQB1*0303 (mumps) [30]. Of interest, the association of the *HLA*-DPB1*0301 allele with the poor response to the rubella vaccine was replicated in a recent GWAS that investigated around five million variants in 1000 healthy individuals vaccinated with the MMR vaccine [33].

Furthermore, genes encoding proteins that interact with the measles virus (the wild and vaccine strains) and augment their entry into host cells were studied in the context of MMR vaccinomics. The most prominent genetic marker from this group of genes was *rs2724384* in the *CD46* gene, which was associated with low antibody response to MMR vaccination in both a candidate gene study on 764 individuals [47] and a GWAS on 2872 individuals [46]. While CD46 is preferentially used by the vaccine strain measles virus, ’signaling lymphocyte activation molecule’ (SLAM) interacts more readily with the wild-type strain. In the candidate gene study, two SNPs in SLAM were associated with measles antibody levels, as well as the IFN-γ and IL-10 responses [47]. In addition to CD46, one of the top hits in the GWAS here [46] was in the ’interferon-induced protein 44 like’ (*IFI44L*) gene (*rs273259*). Interestingly, in the adversomic study by Feenstra et al. [7], the A allele of this polymorphism was associated with increased measles-specific antibody titer [46] and increased risk of febrile seizures after the MMR vaccination. The function of *IFFI44L* is still not clear, but it has been demonstrated that it has a type I IFN response against the hepatitis C virus [51] and appears to be involved in innate immunity.

Toll-like receptors and their intracellular signaling molecules have important roles in innate immunity. Ovsyannikova et al. [48] examined associations between polymorphisms in *TLR* family genes and measles-vaccine-specific immune responses in 764 individuals vaccinated with two doses of MMR. Two SNPs in the coding regions of the *TLR2* (*rs3804100*) and *TLR4* (*rs5030710*) genes were associated with increases and decreases in measles-specific antibodies, respectively. Furthermore, the minor allele of a SNP in the *KIAA1542* gene (*rs702966*) was associated with low IFN-γ response [48].

Vitamin A (retinol) and vitamin D and their receptors are essential regulators of immune function, and they have been linked with susceptibility to various viral infections [52], and they have also been studied in the context of MMR vaccine efficiency [37]. In this study, several SNPs in retinoic acid receptor α (*RARB*) were associated with variations in both measles antibodies and cytokine secretion levels, including IL-10, IFN-α, and TNF-α. Furthermore, significant associations were shown between multiple vitamin D receptors (*VDR*) and retinoid X receptor α (*RXRA*) SNPs/haplotypes and measles-specific IL-2, IL-6, IL-10, IFN-α, IFN-γ, IFNλ-1, and TNF-α cytokine secretion [37].

The ’tripartite motif containing’ TRIM proteins that are activated by type I IFNs were recently shown to be important cellular factors for innate immunity and antiviral defense [53]. Apparently, they also have roles in the MMR vaccine response, as associations have been shown between TRIM5 (*rs7122620*) and TRIM25 (*rs205499*) gene polymorphisms and measles-specific antibody levels [38]. In the same study, several SNPs in TRIM5, 22 and 25 were connected with cytokine responses (IFN-γ, IL-2, IL-6, IL-10, TNF-α).

Recently, a GWAS on 1843 individuals identified four polymorphisms in the tumour suppressor gene *WT1* (*rs4986811*, *rs5030172*, *rs5030157*, *rs5030166*) that were associated with decreased rubella-specific IL-6 secretion from peripheral blood mononuclear cells post-MMR vaccine [39]. However, all four of these polymorphisms were identified in a *WT1* transcript variant known to be a target of ’nonsense mediated decay’, which indicates that its functional role in this vaccine response might be questionable.

In addition to genomic studies, whole transcriptome [54] and micro (mi)RNA profiling [55] have revealed interesting potential new vaccinomic markers. Next-generation sequencing of intracellular mRNA and miRNAs (mRNA-Seq) in measles-virus-stimulated B cells and CD4+ T cells from high and low antibody responders to measles vaccine identified vaccinomic markers from various gene ontology groups: plasma cell survival (CD93, IL6, CXCL12), chemokine/cytokine activity, and cell-cell communication/ adhesion/ migration. The predicted targets for the identified B cell-specific miRNAs included the Fc-receptor and several other signalling pathways, as well as pathways related to transcriptional regulation, viral infection, lipid biosynthesis/metabolism, cytoskeletal protein binding, extracellular matrix–receptor interactions, and apoptosis.

In conclusion here, numerous HLA and non-HLA genetic factors that individually or jointly contribute to the variability seen for the humoral responses to the MMR vaccine have been identified. In the future, new approaches and methods will enable the investigation of immune response mechanisms to the measles vaccine on a new multidimensional level, such as vaccinomics, systems biology, GWAS, epitope prediction, and sophisticated bioinformatics/statistical algorithms, [56]. More details of the genes associated or believed to be associated with MMR vaccine responses are given in Table 1, Table 2, Table 3 and Table 4.

### 2.3. Vaccinomics of the Human Papilloma Virus Vaccine

Vaccination against HPV is intended for prevention of HPV-associated cancers. Vaccination is especially efficient against virus infection types 16 and 18, which are responsible for 70% of cervical cancers worldwide, and types 6 and 11, which are responsible for 90% of cases of genital warts. The Centers for Disease Control and Prevention recommends that all boys and girls get two doses of the HPV vaccine at 11 to 12 years old. To prevent HPV infection, there are currently three vaccines used for young girls from the age of 9 years: the bivalent vaccine Cervarix (directed at HPV types 16, 18), the quadrivalent vaccine Gardasil (directed at HPV type 6, 11, 16, 18) and Gardasil 9 (directed at HPV types 6, 11, 16, 18, 31, 33, 45, 52, 58).

Since the HPV vaccines are relatively novel compared to the HBV and MMR vaccines, no vaccinomics studies have investigated the genetic markers of HPV vaccine efficiency to date. However, HLA and non-HLA genes have dominant roles in the initiation of the antiviral response through innate and humoral immunity networks, and they can also contribute to increased risk of the diseases. Leo et al. associated three haplotypes with increased risk for cervical neoplasia after HPV infection: *HLA*-DRB1*15/*HLA*-DQB1*0602/*HLA*-DQA1*0102, *HLA*-B*0702/HLA-C*0702 and HLA-DRB1*0401/HLA-DQA1*0301 [57]. Mainali et al., investigated 191 men infected with HPV, and they identified novel susceptibility genes involved in HPV-16 pathogenesis for genital persistence. The strongest association was for intergenic variant *rs1293153* (between the *AL133335.1* and *DOK5* genes) and *rs405103* (in gene *AC013565.1*) on chromosomes 20 and 15, respectively [58]. Additionally, the genetic variants of transforming growth factor TGFβ1 (T869C, C509T, and G915C) that have important roles in tumour progression and suppression and in immune suppression have been related to HPV-16–positive oropharyngeal cancer, as compared to their wild-type genotypes [59]. This was further supported by Levovitz et al., who reported that *TGFβR1* is significantly overexpressed in oropharyngeal and cervical cancer [60]. Altogether, these studies have shown the importance of the TGFβ1 signalling pathway in HPV tumour progression, and again, this highlights the involvement of the genetic background in HPV vaccine immune responses. These loci might thus represent good candidate genetic markers in vaccinomics and might be a starting point for evidence-based vaccination.

Recently, a critical review with novel insights and expert opinion on personalized HPV vaccination by Chambuso et al. identified two important strategies to personalization of HPV vaccination [61]: (a) identification of clinically important genetic markers for long-term HPV vaccine immune responses; and (b) profiling of host genetic risk factors that can contribute to modifications to long-term immune responses. Moreover, they also proposed some important research topics related to vaccine effectiveness and safety that should be addressed in the near future: the association between ethnic origin and impaired HPV vaccine immune responses; the correlation of post-vaccination serum antibodies with vaccine adverse effects; the variable persistence of immunity in different populations; the recognition of acute and chronic adverse events of HPV vaccination according to variations in host immune response genes and the number of vaccine doses administered [61].

## 3. Adversomics

Vaccines are a pharmaceutical product that is subjected to the strictest of safety protocols, as they are administered to healthy individuals, and in most cases to paediatric populations. Although modern vaccines have excellent safety profiles, some individuals do very rarely experience serious side effects after vaccination. These side effects are so rare that they can only be detected in the post-marketing phase of vaccine testing, when it has been administered to a very large number of individuals. According to the vaccine pharmacovigilance working groups of the Council for International Organisations of Medical Sciences/World Health Organisation [62], vaccine pharmacovigilance is defined as “the science and activities relating to the detection, assessment, understanding and communication of the adverse effects following immunization and other vaccine or immunization-related issues, and to the prevention of unwanted effects of the vaccine immunization”. Investigation of the underlying causes of such events is essential not only to improve vaccine safety, but also to maintain public confidence in vaccination.

### 3.1. Adversomics of the Hepatitis B Vaccine

Generally, the safety of the HBV vaccine has been evaluated as very high [63,64]. However, over the last two decades following the launch of the massive HBV vaccination campaigns worldwide, there have been reports of the onset of several autoimmune diseases after the HBV administration. These have included: multiple sclerosis, optic neuritis, vasculitis, rheumatoid arthritis, systemic lupus erythematosus, and others. Multiple sclerosis and other demyelinating diseases have been investigated the most, and the majority of these studies have indicated no increased risk of multiple sclerosis after HBV vaccination [65]. However, the doubts about the HBV vaccine risks have not been resolved yet, also because there is good theoretical rationale for HBV-vaccine-induced autoimmunity through various mechanisms [66]. Furthermore, numerous studies on animal and human models have demonstrated activation of transient autoimmunity processes after HBV vaccination in terms of the levels of antibodies [67,68] and T-regulatory cells [69].

While a number of genetic markers that can predict HBV vaccine responses are known to date (i.e., for vaccine efficiency), there are very few related to prediction of adverse reactions to the HBV vaccine, as few such adversomics studies have been performed. Indeed, in general, HBV vaccine adversomic studies have been rare and of a more speculative nature. Belloni et al. concluded that there was no statistically significant difference in the levels of auto-antibodies between responders and non-responders for children vaccinated with the HBV vaccine [31]. However, the non-responder children showed a trend towards higher levels of anti-smooth-muscle antibodies (30% vs. 2% for responders). The anti-smooth-muscle antibody-positive non-responders all carried the supratype *HLA*-*C4AQ0*, DRB1*0301, DQB1*02, which has been associated with poor HBV response in other studies [17,18,20] and is a well-known predisposing factor for autoimmune disorders. Belloni et al. speculated that HBV vaccine non-responders who carry the *HLA*-*C4AQ0*, DRB1*0301, DQB1*02 haplotype might thus be more at risk of autoimmune disease [31]. On the basis of the analysis of a series of case reports of various autoimmune diseases, Miller at al. hypothesized that the presence of certain *HLA*-*DRB1* alleles (*01:01, *03:01, *04:01, *13:01, *15:01) that have been identified by others as HBV vaccine response modulators [17,18,19,20] can result in activation of CD8+T cells by HLA-A2–presented HBV surface antigens, which can result in the production of high levels of IFN-ɣ and TNF, and can promote autoimmune processes [32]. On the similar premise, Mormile hypothesized that infants who are non-responsive to HBV will be at risk for autoimmune disease later in life, and implicated SNPs in the genes for IL-18 and IFN-γ as possible markers of this latent autoimmunity [15]. The concept that certain *HLA* and *HLA*-associated alleles and haplotypes might predispose for nonresponse and autoimmunity after HBV vaccination is in part confirmed by a recent whole-genome transcriptomic study that showed that HBV vaccine non-responders had higher pre-vaccination expression levels of pro-inflammatory genes (especially those in the IFN pathways) compared to HBV vaccine responders [70]. More details of the descriptions of the genes associated or believed to be associated with HBV vaccine safety are given in Table 1.

### 3.2. Adversomics of the MMR Vaccine

Fever or elevated body temperature is one of the most frequent adverse events of vaccines. In particular, febrile seizures have been associated with the live-virus vaccines, such as MMR. While numerous studies have investigated the genetic basis of MMR vaccine efficiency, adversomic MMR studies remain scarce. One of the most encompassing was by Feenstra et al., who carried out a series of GWAS that compared the genetic make-up of children who experienced febrile seizures after the MMR vaccine to children with vaccine-unrelated febrile seizures, and to controls without a history of febrile seizures of any kind [7]. Two SNPs in two genes associated with MMR-related febrile seizures were identified: *IFI44L* (*rs273259*: *P* = 5.9 × 10^−12^ vs. controls; *P* = 1.2 × 10^−9^ vs. MMR-unrelated febrile seizures) and *CD46* (*rs1318653*: *P* = 9.6 × 10^−11^ vs. controls; *P* = 1.6 × 10^−9^ vs. MMR-unrelated febrile seizures) [7]. Interestingly, as mentioned earlier, both of these genes were associated with efficiency of seroconversion after the MMR vaccine [46,47]. In the same study by Feenstra et al., three loci were associated with febrile seizures in general, as these loci differed between the controls and the MMR-vaccine-related seizures, as well as the controls and the MMR-vaccine-unrelated seizures, but not between the groups with MMR-vaccine-related and MMR-vaccine-unrelated seizures: *SCN1A* (*rs6432860*), *SCN2A* (*rs3769955*), and *ANO3* (*rs114444506*) [7]. While *SCN1A* and *SCN2A* encode two subunits of a voltage-gated sodium channel and have already been associated with (febrile) seizures, *ANO3* encodes one of the anoctamin proteins that form part of at least two Ca^2+^-activated chloride channels. Feenstra et al. also performed electrophysiological recordings on brain slices from wild-type and ANO3-null rats to investigate the mechanisms of ANO3 involvement in the genesis of febrile seizures. They reported that hippocampal neurons of *ANO3*-null rats showed increased excitability compared to neurons of wild-type rats at both normal and febrile body temperatures [7].

In extremely rare cases, MMR vaccination can lead to severe or chronic infections in children, which sometimes includes measles inclusion-body encephalitis, with the associated mortality rates as high as 10% to 20% [71]. This typically occurs in immunocompromised individuals, and is mostly due to inborn errors of immunity. T cell deficiencies, such as severe combined immune deficiency, have been associated with severe outcomes after MMR vaccine administration. However, such cases are particularly rare in clinical practice, as most patients with severe combined immune deficiency are diagnosed very early in life, and before administration of their first MMR vaccine dose [71]. Interestingly, in DiGeorge syndrome, where patients have mild-to-moderate T cell lymphopenia but intact T cell function, no severe outcomes after MMR vaccination have been reported [71].

On the other hand, inborn errors of type I IFN immunity might be more important in this clinical setting considering severe infection after the MMR vaccine, as these conditions have milder presentation and are usually diagnosed later in life. Interestingly, an ontology-based literature mining study identified IFN-γ as one of the top ranking genes in both vaccine and fever networks [72]. There have been a handful of case reports that have described mutations in genes in the IFN networks as a cause for severe adverse reactions to the MMR vaccine. In these case reports, mutations in the following genes have been associated with severe measles infection (and in some cases death) after MMR vaccine administration in otherwise healthy children without overt signs of immune deficiency: *STAT1* [40], *STAT2* [41,42,43], *INFAR1* [35], *INFAR2* [36], *IRF7* [44] and *IRF9* [45]. More detailed descriptions of the genes associated or believed to be associated with MMR vaccine safety are given in Table 2, Table 3 and Table 4. Of note, the study that used ontology-based literature mining identified several other genes that have not yet been investigated in the scope of MMR vaccine adversomics as high ranking in both vaccine and fever networks (i.e., *IL1B*, *TNF*, *IL-6*, and *CD8A*), and in only fever networks (*HSPA1A*, *NFKB1*, *IL-8*, *IL-2*, *MEFV*, *MAPK1*, *POMC*, *CD4*, and *IL-10*), and in only vaccine networks (*CSF2*, *IL7R*, *ERVWE1*, *APC*, *MC4R*, *IL1R1*, and *TLR2*) [72]. In our opinion, these genes represent good candidates for future adversomic studies of the MMR vaccine.

### 3.3. Adversomics of the Human Papilloma Virus Vaccine

The HPV vaccines are considered safe and efficient. Since the initial approval of Gardasil by the US Food and Drug Administration in 2006, Gardasil, Gardasil 9 and Cervarix have all been through extensive safety testing in many clinical trials, which have included more than 29,000, 15,000 and 30,000 females and males, respectively [73]. As with other vaccines, the commonly reported side effects are mild. However, there have been a few well-described clinical cases of exacerbation of autoimmune–rheumatic conditions after HPV vaccination, with particular reference to manifestation of systemic lupus erythematosus, and systemic-lupus-erythematosus-like disease [74,75]. The cases that have been reported have included females aged 13 to 32 years with a personal or family history of various autoimmune–rheumatic conditions. This again highlights the importance of the genetic predisposition of the individual, with an environmental factor as a second hit to provoke autoimmune diseases. At the same time, it cannot be ignored that women with systemic lupus erythematosus show higher prevalence for HPV infection compared to healthy women, as the disease itself weakens the natural and humoral responses to infections [74,76]. Risk-benefit considerations need to be taken into account by physicians for each individual case, based on the depth of the anamnesis, the family history, and the genetic background prior to vaccination.

The recent literature also includes reviews of ’HPV vaccination syndrome’ as a model for pathogenesis for fibromyalgia [77,78,79]. Fibromyalgia is defined as chronic widespread pain, which is commonly accompanied by joint stiffness, fatigue and sleep disturbance, along with cognitive, autonomic and sensory dysfunction. GWAS studies have identified two nonsense mutations here [80,81]: W32X in *C110rf40* and Q100X in *ZNFJJ*. These have been associated with high levels of the cytokines that are involved in inflammation exacerbation.

### 3.4. Biological Sex Differences in Adversomics

It is now widely accepted that biological sex differences influence the type and strength of the immune response to a pathogen infection or in vaccination. Sex differences (e.g., sex, age, reproductive cycle, hormones, and genetic factors) affect all parts of the immune system—innate, humoral, and cellular response [82,83]. In general, compared to men and relevant for vaccination, women have a higher antibody response, an increased number of B cells, an increased number and activity of Treg and Tc cells and an increased number of macrophages with a higher release of pro-inflammatory cytokines. Despite the qualitative and quantitative senescence of the immune system, these differences persist from childhood to old age. For many vaccines, including HBV, HPV studies in women show increased antibody titres and more adverse reactions than men [84,85,86]. Another reason for a more intense immune response in women is the X chromosome, which contains a high density of immune-related genes. Although the second copy of the X chromosome in women is inactivated through the mechanisms of epigenetics, parts of it can be reactivated or skewed X chromosome deactivation occurs [87,88]. Since both topics are highly relevant for the efficacy and side effects of the vaccine, for further details please refer to Flanagan et. al. [82] and Klein et. al. [89] and the references contained therein.

Despite all this knowledge, not many clinical vaccination studies take immunological differences into account. Data are usually combined, analyses by sex and gender are not considered or are not applicable due to the inequality of the samples. A rational design was proposed by Klein S.L. and Pekosz A. for influenza vaccination, taking into account biological sex differences [90]. In practice, the ultimate goal would be to reduce inflammation and side effects in women while maintaining efficient seroconversion; reducing vaccine doses in women would also mean greater availability of the vaccine within the population. At present, such a “male and female” vaccination approach seems to be a long way off. Many scientists and clinicians agree that such a rational design could have added value in terms of efficacy and adverse events; we also agree that it would be an important step forward in the field of personalized medicine.

### 3.5. Challenges and Future Perspectives in Adversomics

The lack of studies in the field of adversomics is at least in part due to the several challenges in the study design. The main problem that the researchers face is the question, which side effects of the vaccine are relevant to its safety and thus worth studying.

Mild side effects are not life threatening and can be an indicator of the efficient immune response to the vaccine, leading to the conclusion that they are not a priority in adversomic studies. On the other hand, even mild side effects can produce anxiety in patients and/or their parents and thus lead to the discontinuation of the vaccination, causing low vaccination rates. From this point of view, the determination of genetic markers of mild side effects of childhood vaccines might be rational. The ability to predict even mild side effects prior to vaccination might alleviate parental fears and lead to the increased trust in vaccination programmes and health system in general. Additional problem in studying mild side effects is that they tend to be underreported by health providers, leading to problems in cohort stratification. Therefore, in order to obtain relevant results the full cooperation of physicians in reporting even mild side effects of vaccinations is of the key importance.

On the other hand, serious side effects are usually promptly reported and the rationale for the identification of genetic markers predicting such side effects is clear. However, because serious side effects of vaccines are very rare in the population, the major challenge in such studies is collecting the sufficient number of cases to reach high enough statistical power. This might be overcame by using multicentric international approach, but unfortunately, different countries have heterogeneous systems and databases of reporting vaccine side effects, which leads to problems in the identification of the relevant cases. What is needed is the international database for vaccine side effects registration, which would enable relevant adversomic studies.

In conclusion, to enable the development of the field of vaccine adversomics the international database for reporting vaccine side effects accessible to researchers globally is needed, as well as full cooperation of health providers in reporting all vaccine side effects.

## 4. Future Perspectives on Vaccinomics and Adversomics of a COVID-19 Vaccine

At least eight types of vaccines are under development against SARS-CoV-2: virus vaccines (inactivated, weakened); viral vector vaccines (replicating, non-replicating); protein-based vaccines (protein subunit, virus-like particles); and nucleic acid vaccines (DNA, RNA). As of 11 May 2020, eight SARS-CoV-2 candidate vaccines were being tested on humans, and among these, the ’genetic immunization’ with DNA or RNA vaccines shows potentially the most promising results. This approach is new for human use, and although there are several (non-COVID-19) DNA vaccines available for veterinary use, none of these have been approved for humans. However, important design knowledge has been obtained from several previous threats of such virus diseases, including from SARS-CoV in 2003, H5N1 avian influenza in 2005, H1N1 pandemic influenza in 2009, and Zika virus in 2016. This allowed DNA (Moderna) and RNA (mRNA; Inovio) SARS-CoV-2 vaccines to already be tested in clinical trials in April 2020. DNA-based and RNA-based vaccines should overcome some important safety worries and unwanted side effects as these do not contain any virus; instead, they include only the genetic material of the virus immunogenic protein (e.g., the ’spike protein’ of SARS-CoV-2), which is inserted into human cells. After successful uptake of DNA, the human cell produces viral proteins that prompt an immune response, and thus protect against infection. Yu et al. have recently published promising results for their SARS CoV-2 DNA vaccine testing on 35 rhesus macaques [91]. The most significant reduction in viral replication was observed in the upper and lower respiratory tract, with full-length S immunogen after three doses of the vaccine (zero, one, and five weeks). The vaccinated animals developed humoral and cellular responses that included neutralizing antibody titres with comparable magnitude to the antibody titres in convalescent macaques and convalescent humans. A recent study on 149 COVID-19 convalescent individuals has also reported encouraging results; namely, even though the plasma titres of the neutralizing antibodies against the receptor binding domain of the SARS-CoV-2 spike protein were relatively low in most patients, they were present in all of the individuals tested, which suggests that a vaccine designed to elicit these antibodies should be broadly effective [92].

In our opinion, personalized approaches to SARS-CoV-2 vaccine development will be even more important than for other vaccines, as profound differences in the patient responses to SARS-CoV-2 infection have been reported since the early stages of the COVID-19 pandemic. While in most infected individuals relatively mild respiratory symptoms have been noted, a certain subpopulation of patients undergoes a severe cytokine storm that can lead to multi-organ failure and high levels of mortality. Several environmental and demographic factors have been attributed to these interindividual differences in COVID-19 severity, but there are some indications that genetic factors have an important role here. Considering SARS-CoV-2 vaccine development, it should be noted that patients who have severe reactions to the infection might be more at risk for adverse reactions to the vaccine (i.e., cytokine storm). Conversely, individuals with asymptomatic presentation might be potential vaccine non-responders. Thus, the genetic markers identified as predictors of COVID-19 severity should be taken into account during the vaccine development and its later administration.

In May 2020, an important international initiative called the ’COVID-19 Host Genetics Initiative’ was started [93], which aims to determine host genetic factors that can explain interindividual differences in responses to SARS-CoV-2 infection, with particular focus on HLA genes. At time of writing (end of September 2020), 216 studies had joined the initiative, which is both retrospective and prospective in nature, and mostly centred on Europe and USA, with participation still expanding. Retrospective collections are typically biobanks that already include significant genetic data and active connections to health systems, while prospective collections have recently started to directly obtain consent from incoming COVID-19 patients. Retrospective collections enable rapid development of genetic studies on susceptibility and severity, while prospective approaches bring important additional opportunities not only for deeper DNA studies, but also for potentially informative viral and antibody profiling and epitope mapping experiments that can be implemented in vaccine development [93].

The angiotensin-converting enzyme-2 gene, *ACE2,* has been hypothesized to have an important role in COVID-19 severity, as it was identified early on as a facilitator of the SARS-CoV-2 virus entry into cells. The *ACE2* product, angiotensin-converting enzyme-2, has been shown to be the receptor for both SARS-CoV-2 virus and the NL63 human respiratory coronavirus. A recent study analyzed all of the 1700 variants in the *ACE2* gene from the China Metabolic Analytics Project and 1000 Genomes Project databases. No direct evidence was found for coronavirus S-protein binding-resistant ACE2 mutants in different populations [94]. However, East Asian populations have much higher allelic frequencies of genetic variants associated with higher ACE2 expression in tissues, which indicates differential susceptibility to SARS-CoV-2 in different populations [94].

**Figure 1:**
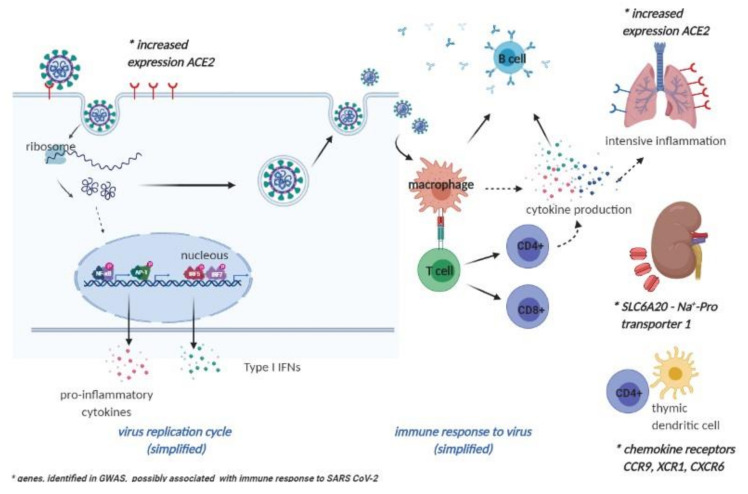
Genes identified in genome association studies (GWASs), possibly related to the immune response to SARS-CoV-2. (Adapted from “Coronavirus Replication Cycle”, by BioRender.com (2020). Retrieved from https://app.biorender.com/biorender-templates).

In contrast, an agnostic approach has revealed several genes of interest that might have roles in SARS-CoV-2 infection responses. A meta-analysis of two GWASs that involved patients with severe COVID-19 (as 835 and 775 individuals in the Italian and Spanish cohorts, respectively) and mostly non-infected controls from the general population (as 1255 and 950 individuals in the Italian and Spanish cohorts, respectively), revealed two critical loci that were responsible for severe COVID-19 presentation: 3p21.31 and 9q34.2. The 9q34.2 locus is associated with the ABO blood groups, which thus confirms the previous anecdotal observations of differential SARS-CoV-2 responses in individuals with different blood groups. In this meta-analysis, blood group A was associated with more severe disease, while blood group 0 was protective. However, the latest multi-institutional study on 7648 COVID-19–positive samples did not support the association of blood type with risk of intubation or death in COVID-19 patients, although it confirmed that blood type O was less common for SARS-CoV-2 infection [95]. Considering the first locus, 3p21.31, six genes have been defined for this area: *SLC6A20*, *LZTFL1*, *CCR9*, *FYCO1*, *CXCR6*, and *XCR1* [96], some of which appear directly or indirectly connected to SARS-CoV-2 responses (Figure 1). *SLC6A20* encodes sodium–proline transporter 1, which can functionally interact with ACE2, the SARS-CoV-2 cell-surface receptor. *CCR9*, *XCR1* and *CXCR6* encode chemokine receptors, the last of which regulates the specific location of lung-resident memory CD8 T cells throughout sustained immune responses to airway pathogens [96]. The functions of these other two genes at the 3p21.31 locus include protein trafficking (*LZTFL1*) and autophagy (*FYCO1*).

Previously mentioned sex differences in immune response and hormonal status should not be neglected also in the infection with SARS-CoV2. In many states, reporting sex-disaggregated data, COVID-19 with severe disease symptoms and higher death rates are seen among men [97,98]. Recently, Takahashi T et al. provided us with findings, that poor T cell response is associated with worse disease outcome in male patients, but not in female patients. Additionally, higher levels of innate immune cytokines (Il-8 and IL-18) at the disease baseline and during the course of the disease (chemokine CCL5) were associated with worse disease progression in males. Results of this study have shown equality in humoral but diversities in cellular immune response as well as the levels of the effector molecules. The authors suggested that vaccines and therapies increasing T cell immune response to SARS-CoV-2 might be appropriate for male patients, and those dampening the innate immune activation in the early stage of the disease for females [99].

## 5. Conclusions

Vaccination has been one of the most successful public health interventions in human history, as it continues to save millions of lives every year. However, the effectiveness and safety of some vaccines is limited by the heterogeneity of the responses among different individuals and across different populations. Heterogeneity of vaccine responses is a multifactorial trait, which results from a combination of environmental and genetic factors. Several environmental factors that contribute to this variation have been identified, such as age, gender, ethnicity, body-mass index and health, and smoking status, as well as dose, route of administration and quality of storage of the vaccine. Twin studies have also identified high levels of heritability of vaccine responses. Thus, identification of the genetic factors that modulate responses to vaccine administration is of the utmost importance for the development of safe and effective vaccines, to increase the public trust in vaccines, and consequently to increase the background vaccination rates.

## Figures and Tables

**Table 1:** Details of HLA genes associated with HBV and MMR vaccine efficacies and safety. HLA: human leukocyte antigen; HBV: Hepatitis B virus; MMR: measles, mumps, and rubella.

Gene	Gene Description	Genomic Location	Function of Gene Product	Associated Disease Phenotypes–Not Vaccine Related [28]	Studies on Associated Vaccinomic Phenotypes		Studies on Associated Adversomics Phenotypes
					HBV	MMR	HBV
*HLA-DRB1*	Major histocompatibility complex, class II, DR β 1	6p21.32	HLA class II β chain paralogue, presenting peptides derived from extracellular proteins	Sarcoidosis, multiple sclerosis, rheumatoid arthritis, autism/schizophrenia	[17,18,19,20,21,23,25]	[29,30]	[31,32]
*HLA-DQB1*	Major histocompatibility complex, class II, DQ β 1	6p21.32	HLA class II β chain paralogue, presenting peptides derived from extracellular proteins	Celiac disease, multiple sclerosis, Creutzfeldt-Jakob disease, systemic lupus erythematosus, autism/schizophrenia	[17,18,19,20,22,25]	[30]	[31]
*HLA-DPB1*	Major histocompatibility complex, class II, DP β 1	6p21.32	HLA class II β chain paralogue, presenting peptides derived from extracellular proteins	Chronic beryllium disease, Wegener granulomatosis, chronic hepatitis B infection	[24,25]	[30,33]	None
*HLA-B*	Major histocompatibility complex, class I, B	6p21.33	HLA class I heavy chain paralogue, presenting peptides derived from the endoplasmic reticulum lumen	Behcet disease, pulmonary arterial hypertension, toxic epidermal necrolysis, spondyloarthropathy, Stevens-Johnson syndrome, Takayasu arteritis	None	[29,30]	None
*HLA-DQA1*	Major histocompatibility complex, class II, DQ α 1	6p21.32	HLA class II α chain paralogue, presenting peptides derived from extracellular proteins	Myasthenia gravis, celiac disease, idiopathic achalasia	None	[29,30]	None
*HLA-A*	Major histocompatibility complex, class I, A	6p22.1	HLA class I heavy chain paralogue, presenting peptides derived from the endoplasmic reticulum lumen	Cancers, birdshot chorioretinopathy, myelodysplastic syndrome, toxic epidermal necrolysis	None	[30]	None
*HLA-DPA1*	Major histocompatibility complex, class II, DP α 1	6p21.32	HLA class II α chain paralogue, presenting peptides derived from extracellular proteins	Granulomatosis with polyangiitis	None	[30]	None

**Table 2:** Details of genes encoding cytokines and their receptors associated with HBV and MMR vaccine efficacies and safety.

Gene	Gene Description	Genomic Location	Gene Product Function	Associated Disease Phenotypes–Not Vaccine Related [28]	Studies on Associated Vaccinomic Phenotypes		Studies on Associated Adversomics Phenotypes
					HBV	MMR	MMR
*TNF*	Tumour necrosis factor	6p21.33	Cytokine, regulation of cell proliferation, differentiation, apoptosis, lipid metabolism, coagulation	Asthma, malaria susceptibility, migraine	None	[34]	None
*TNFRSF1A*	TNF receptor superfamily member 1A	12p13.31	TNF receptor, cell survival, apoptosis, and inflammation	Intermittent hydrarthrosis, multiple sclerosis, familial periodic fever, TNF receptor 1 associated periodic syndrome	None	[34]	None
*IL-6*	Interleukin 6	7p15.3	Cytokine, inflammation, and maturation of B cells	Arteriovenous malformations of the brain, diabetes mellitus type I, Crohn’s disease, Kaposi sarcoma, juvenile rheumatoid arthritis, juvenile idiopathic arthritis	None	[34]	None
*IL-1β*	Interleukin 1β	2q14.1	Cytokine, mediator of inflammatory responses	Gastric cancer risk (*Helicobacter pylori* induced), periodontal disease	[27]	[34]	None
*IL-13*	Interleukin 13	5q31.1	Cytokine, B cell maturation, promotion of IgE isotype switching of B cells	Asthma, allergic rhinitis	[27]	None	None
*IL-4*	Interleukin 4	5q31.1	Cytokine, B cell activation, IgE secretion	Allergic bronchopulmonary aspergillosis, schistosomiasis	[27]	None	None
*IL4RA*	Interleukin 4 receptor	16p12.1	Receptor for IL-4 and IL-13, promotes Th2 differentiation	Atopy, human immunodeficiency virus -1 resistance	[27]	None	None
*IL2RA*	Interleukin 2 receptor subunit α	10p15.1	Part of IL-2 receptor, T cell-mediated immune responses	Diabetes mellitus type I, immunodeficiency, juvenile idiopathic arthritis	None	[34]	None
*IL2RB*	Interleukin 2 receptor subunit β	22q12.3	Part of IL2 receptor, T cell-mediated immune response	Juvenile idiopathic arthritis, immunodeficiency	None	[34]	None
*IFNB1*	Interferon β1	9p21.3	Cytokine, defense against viral infections, cell differentiation, anti-tumor defense	Multiple sclerosis	None	[34]	None
*INFAR1*	Interferon α/β receptor subunit 1	21q22.11	One of two chains of a receptor for INFα and INFβ, activation of receptor stimulates Janus protein kinases, which phosphorylate STAT1 and STAT2	Hepatitis C susceptibility, measles susceptibility	None	None	[35]
*INFAR2*	Interferon α/β receptor subunit 2	21q22.11	One of two chains of a receptor for INFα and INFβ, activation of receptor stimulates Janus protein kinases, which phosphorylate STAT1 and STAT2	Measles susceptibility, immunodeficiency 45	None	None	[36]

**Table 3:** Details of genes encoding transcription factors and transcription regulators associated with MMR vaccine efficacy and safety.

Gene	Gene Description	Genomic Location	Gene Product Function	Associated Disease Phenotypes–Not Vaccine Related [28]	Studies on Associated Vaccinomic Phenotype: MMR	Studies on Associated Adversomics Phenotypes: MMR
*RARB*	Retinoic acid receptor β	3p24.2	Nuclear transcriptional regulator, binds retinoic acid	Matthew-Wood syndrome, microphthalmia, diaphragmatic hernia	[37]	None
*RXRA*	Retinoid X receptor α	9q34.2	Nuclear receptor, involvement in retinoic-acid-mediated gene activation	Colon adenoma, recessive dystrophic Epidermolysis bullosa	[37]	None
*VDR*	Vitamin D receptor	12q13.11	Ligand-inducible transcription factor, also a receptor for the secondary bile acid, lithocholic acid	Hypocalcaemic vitamin D-resistant rickets, osteoporosis, vitamin D-dependent rickets (type 2a)	[37]	None
*TRIM25*	Tripartite motif-containing 25	17q23.1	Transcription factor, mediates estrogen actions in breast cancer	Influenza, swine influenza	[38]	None
*WT1*	WT1 transcription factor	11p13	Transcription factor, development of urogenital system, tumor suppressor gene	Gonadal dysgenesis, cancers, aniridia, Denys-Drash syndrome, Frasier syndrome, genetic steroid-resistant nephrotic syndrome, Meacham syndrome, ulcerative colitis, Wilms tumor, aniridia, genitourinary anomalies, and retardation syndrome	[39]	None
*STAT1*	Signal transducer and activator of transcription 1	2q32.2	Transcription activator, mediates expression of a variety of genes, which is thought to be important for cell viability in response to pathogens, can be activated by IFNα and IFNγ	Autoimmune enteropathy and endocrinopathy, immunodeficiency 31A, 31B, and 31C, susceptibility to viral and mycobacterial infections	None	[40]
*STAT2*	Signal transducer and activator of transcription 2	12q13.3	Transcription activator, in response to IFN, forms a complex with STAT1 and ISGF3G	Immunodeficiency 44, primary immunodeficiency with post-measles-mumps-rubella vaccine viral infection	None	[41,42,43]
*IRF7*	Interferon regulatory factor 7	11p15.5	Transcriptional activation of virus-inducible cellular genes, including IFNβ chain genes	Immunodeficiency 39	None	[44]
*IRF9*	Interferon regulatory factor 9	14q12	Transcription factor, mediates signaling of IFNα and IFNβ, IRF9/ISGF3G associates with the phosphorylated STAT1:STAT2 dimer to form ISGF3 transcription factor	Immunodeficiency 65, susceptibility to viral infections	None	[45]

**Table 4:** Details of other genes associated with HBV and MMR vaccine efficacies and safety.

Gene	Gene Description	Genomic Location	Gene Product Function	Associated Disease Phenotypes–Not Vaccine Related [28]	Studies on Associated Vaccinomic Phenotypes		Studies on Associated Adversomics Phenotypes
					HBV	MMR	MMR
*CD46*	CD46 molecule	1q32.2	Cofactor activity for inactivation of complement components C3b and C4b by serum factor I	Atypical hemolytic uremic syndrome with complement gene abnormality, hemolysis, elevated liver enzymes, and a low platelet count syndrome	None	[46,47]	[7]
*BTNL2*	Butyrophilin-like 2	6p21.32	MHC-II-associated, transmembrane protein, negative T cell regulator	Sarcoidosis, multiple sclerosis, autism/schizophrenia	[22,23,25]	None	None
*SLAM*	Signaling lymphocytic activation molecule family member 1	1q23.3	Self-ligand receptor of signaling lymphocytic activation molecule, modulating the activation and differentiation of a wide variety of immune cells	Measles susceptibility, subacute sclerosing panencephalitis	None	[47]	None
*IFI44L*	Interferon-induced protein 44 like	1p31.1	Unknown, shown a low antiviral activity against hepatitis C virus	Lymph node tuberculosis, Aicardi-Goutieres syndrome	None	[46]	[7]
*TLR4*	Toll-like receptor 4	9q33.1	Pathogen recognition and activation of innate immunity	Behcet’s disease	None	[48]	None
*TLR2*	Toll-like receptor 2	4q31.3	Pathogen recognition and activation of innate immunity	Leprosy susceptibility, tuberculosis susceptibility	[27]	[48]	None
*KIAA1542*	PHD and ring finger domains 1	11p15.5	Protein domain-specific binding and RNA polymerase binding	Systemic lupus erythematosus	None	[48]	None
*TRIM5*	Tripartite motif-containing 5	11p15.4	E3 ubiquitin-ligase, may have role in retroviral restriction	Rubella susceptibility, immune deficiency disease	None	[38]	None
*TRIM22*	Tripartite motif-containing 22	11p15.4	Mediates interferon antiviral effects	Rubella susceptibility, hepatitis B susceptibility	None	[38]	None
*SCN1A*	Sodium voltage-gated channel α subunit 1	2q24.3	Sodium channel α subunit, regulates sodium exchange between intracellular and extracellular spaces, generation of action potentials in muscle cells and neurons	Dravet syndrome, early infantile epileptic encephalopathy 6, familial or sporadic hemiplegic migraine, generalized epilepsy with febrile seizures plus (type 2), Lennox-Gastaut syndrome	None	None	[7]
*SCN2A*	Sodium voltage-gated channel α subunit 2	2q24.3	Sodium channel α subunit, regulates sodium exchange between intracellular and extracellular spaces, generation of action potentials in muscle cells and neurons	Benign familial infantile epilepsy, benign familial neonatal-infantile seizures, Dravet syndrome, early infantile epileptic encephalopathy, generalized epilepsy with febrile seizures-plus, West syndrome	None	None	[7]
*ANO3*	Anoctamin 3	11p14.2	Membrane protein, Ca^2+^-activated chloride channel	Cranio-cervical dystonia with laryngeal and upper-limb involvement	None	None	[7]
